# SPIKE – a database, visualization and analysis tool of cellular signaling pathways

**DOI:** 10.1186/1471-2105-9-110

**Published:** 2008-02-20

**Authors:** Ran Elkon, Rita Vesterman, Nira Amit, Igor Ulitsky, Idan Zohar, Mali Weisz, Gilad Mass, Nir Orlev, Giora Sternberg, Ran Blekhman, Jackie Assa, Yosef Shiloh, Ron Shamir

**Affiliations:** 1 The David and Inez Myers Laboratory for Genetic Research, Department of Molecular Genetics and Biochemistry, Sackler School of Medicine, Tel Aviv University, Tel Aviv 69978, Israel; 2 School of Computer Science, Tel Aviv University, Tel Aviv 69978, Israel; 3 Department of Human Genetics, University of Chicago, Chicago, IL 60637, USA

## Abstract

**Background:**

Biological signaling pathways that govern cellular physiology form an intricate web of tightly regulated interlocking processes. Data on these regulatory networks are accumulating at an unprecedented pace. The assimilation, visualization and interpretation of these data have become a major challenge in biological research, and once met, will greatly boost our ability to understand cell functioning on a systems level.

**Results:**

To cope with this challenge, we are developing the SPIKE knowledge-base of signaling pathways. SPIKE contains three main software components: 1) A database (DB) of biological signaling pathways. Carefully curated information from the literature and data from large public sources constitute distinct tiers of the DB. 2) A visualization package that allows interactive graphic representations of regulatory interactions stored in the DB and superposition of functional genomic and proteomic data on the maps. 3) An algorithmic inference engine that analyzes the networks for novel functional interplays between network components.

SPIKE is designed and implemented as a community tool and therefore provides a user-friendly interface that allows registered users to upload data to SPIKE DB. Our vision is that the DB will be populated by a distributed and highly collaborative effort undertaken by multiple groups in the research community, where each group contributes data in its field of expertise.

**Conclusion:**

The integrated capabilities of SPIKE make it a powerful platform for the analysis of signaling networks and the integration of knowledge on such networks with *omics *data.

## Background

Our realization of the complexity of signaling networks that regulate cellular physiology is growing commensurate with the rapid growth in biological knowledge. It is now clear that biological pathways that govern cellular development and responses to environmental challenges are not linear, parallel and independent, but rather form an intricate web of interlocking processes tightly controlled by various logics of positive and negative feedback loops [1,2]. Given this high degree of complexity, it is essential to develop computational means for processing, presenting and analyzing cellular signaling networks. However, at the present time most biological knowledge resides as free text in archives of scientific journals. Before this knowledge can be processed by computers, it has to be transformed into symbolic form using highly structured languages. The need to represent biological knowledge in a formal language within electronic knowledge-bases (KBs) is well recognized and several ontologies have been defined and electronic repositories have been established in recent years. Many of them (e.g., EcoCyc [3], WIT [4]) focus on metabolic pathways in lower organisms, which at present are the most characterized pathways. KBs are also being developed for signal transduction pathways in higher eukaryotes together with supporting network visualization packages (e.g., KEGG [5], Reactome [6], aMAZE [7,8], BIND [9], PATIKA [10] and CellDesigner [11]).

Biological KBs are also becoming essential to the analysis of data obtained by high-throughput functional genomic and proteomic technologies. For example, when the effect of a certain perturbation on the cellular transcriptome is examined, hundreds of genes typically respond. One way to understand the biological meaning of the observed response is to systematically integrate these results with current biological knowledge and then search for pathways that are significantly enriched for responding genes. GenMapp [12], KEGG [5], and Cytoscape [13] are examples of tools that provide such capabilities in various forms.

We are developing SPIKE (Signaling Pathway Integrated Knowledge Engine) as a tool to help researchers integrate, visualize, interpret and share existing and novel information on cellular signaling networks, and to boost the biological interpretation of wide-scale '*omic*' datasets. SPIKE's DB already contains extensive and highly curated data on signaling pathways in human cells related to DNA damage and other stress responses, cell cycle checkpoints, apoptosis, and innate immunity signaling, in addition to data from massive screenings for human protein-protein interaction. The main feature that distinguishes SPIKE from other extant signaling KBs is its design and implementation as a community tool. Our vision is that the manually curated tier of the DB will be populated by a distributed and highly collaborative effort undertaken by multiple groups in the research community, where each group contributes data in its field of expertise. To meet this goal, both the modeling scheme used in SPIKE for the representation of signaling pathways and the process of data upload by end users are kept very simple, making SPIKE a highly flexible and agile platform for storing, sharing and analyzing data on cellular networks.

## Implementation

### Overall architecture

**Figure 1:**
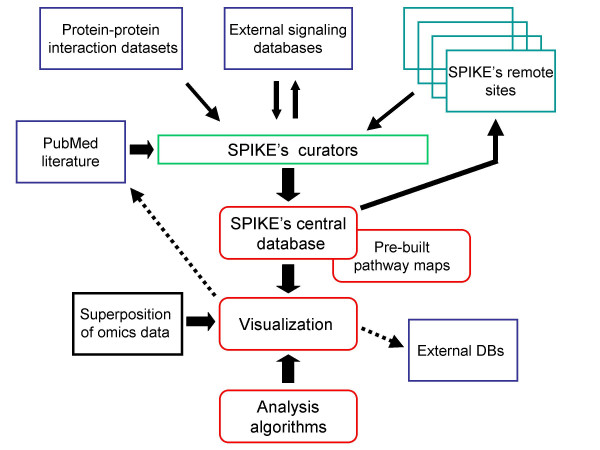
**An overview of SPIKE**. This scheme describes the key components of the system and information flow. SPIKE's three main software components are: 1) A DB of signaling pathways, containing, in distinct tiers, carefully ascertained information uploaded by SPIKE users and supervised by the curators, data from external signaling DBs (e.g., KEGG, Reactome, IntAct), and wide-scale human protein-protein interaction datasets [21–24]. In SPIKE's decentralized architecture, a copy of the DB is installed along with the software in each participating research lab, and these remote site DBs are periodically synchronized with the central DB. The information stored in the SPIKE DB can be readily used by other KBs. 2) A visualization package that allows interactive representation of selected regulatory interactions from the DB, dynamic layout and navigation through the displayed networks, and superposition of high-throughput genomic and proteomic data. Information displayed in the maps is linked to external DBs (e.g., Entrez Genes, PubMed). Prearranged maps for key signaling networks (e.g., p53, apoptosis, cell cycle, ATM, MAPK) were built and are posted at SPIKE website. 3) An algorithmic engine that performs various network analyses, aimed at discovering novel functional interplays between network components.

SPIKE is composed of three main software components: 1) A database (DB) of biological signaling pathways with an interface that supports data uploading and querying by multiple end-users. Carefully curated information from the literature as well as data from large public sources constitute distinct tiers of the DB. SPIKE DB is implemented using MySQL as the DB management system (DBMS). 2) A Java-based visualization package that allows interactive graphic representations of regulatory interactions stored in the DB, dynamic layout and navigation through the networks, and superposition of high-throughput genomic and proteomic data on the displayed maps. 3) An algorithmic inference engine that analyzes the networks for novel functional interplays between network components, and enhances the analysis of *omic *datasets (Figure 1).

### Modeling scheme

SPIKE uses a formal modeling scheme in which information on signaling pathways is summarized in a structured language amenable to computerized manipulation and analysis. The scheme is based upon three fundamental requirements: First, it should be of sufficient expressive power to capture information on most aspects of regulatory pathways. Second, it should focus on signaling rather than metabolic pathways, and on the regulatory interplays maintained between network components (the biochemical mechanisms by which the regulatory effects are elicited are of secondary importance in our model). Third, the scheme should be kept simple in order to ease data input by the SPIKE user community. Such input is crucial as our long-term goal is to have the manually curated tier of the DB populated primarily by the users' community.

**Figure 2:**
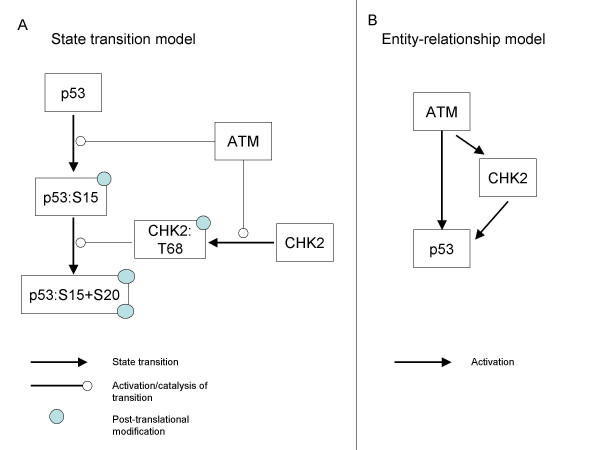
**SPIKE modeling scheme**. State transition scheme vs. entity-relationship scheme. (A). The 'state transition' scheme regards different post-translational modified versions of a protein as separate entities (or as different 'states' of a protein), visually represents them as distinct nodes in the map, and aims at tracing the transition between these states. (B). In contrast, the 'entity-relationship' scheme views all these entities as one protein, and focuses on the regulatory effects between different proteins within a signaling network. In a state transition scheme, representation of signaling pathways could become extremely complex due to combinatorial growth of possible states. An entity-relationship scheme is much simpler, having all these states represented by only one entity, but at the price of not taking into account temporal order constraints on the state transition reactions in cases where such constraints exist.

With these considerations in mind, we adopted (using the terminology of Kitano et al. [11]) an *'entity-relationship*' scheme over a *'state transition*' one. The 'state transition' scheme regards different post-translational modified versions of a protein as separate entities (or as different 'states' of a protein), visually represents them as distinct nodes in the map, and seeks to trace the transition between these states. The 'entity-relationship', on the other hand, does not distinguish between different modified states of a protein and does not look for such state transitions; it focuses, rather, on the regulatory effects maintained between different proteins within a signaling network (Figure 2A–B). In a state transition scheme (adopted, for example, by Reactome [6], PATIKA [10], CellDesigner [11]), representation of signaling pathways could become extremely complex due to combinatorial growth of possible states (for example, a protein that can be modified on only 5 residues has 2^5 ^= 32 different states). An entity-relationship scheme (adopted, for example, by KEGG [5] and Kohn's maps [14,15]) is much simpler, having all these states represented by only one protein entity, but at the price of not accounting for possible temporal order constraints on the state transitions (e.g., activation of a protein by its phosphorylation on residue B can occur only after it is first phosphorylated on residue A).

The entity-relationship scheme we designed for SPIKE is based on two basic types of objects: "*biological entities*" and "*relations*".

#### Biological entities

SPIKE's modeling scheme comprises four types of biological entities:

##### a. Gene/RNA/Protein

Genes (and their respective RNA and protein products) are the primary atomic elements of the scheme. To avoid ambiguities, only characterized human genes that are assigned a formal designation by the Human Genome Nomenclature Committee (HGNC) are included in SPIKE's gene space. These objects are uniquely identified by their Entrez Gene IDs [16]. For simplicity, we decided not to define distinct objects for genes, their transcribed RNAs and translated proteins, and their modifications. See below a discussion of scheme limitations. Non-protein coding genes (e.g., genes encoding for rRNAs, tRNAs and microRNAs) are also included in this entity type.

##### b. Family

A family is a group of isoform genes (encoded by distinct genomic loci) with high sequence homology and whose encoded proteins share most of their biological activities. Well known examples are the JNK family, which includes the JNK1, JNK2 and JNK3 proteins (whose official names are MAPK8, MAPK9 and MAPK10), and the p38 family, which is comprised of four p38 isoforms (officially designated as MAPK11–14).

##### c. Complex

A complex is a group of proteins (or protein families) that carry out a specific function only when associated with their complex mates. An example is the DNA-damage sensor MRN complex, which is composed of the proteins MRE11, RAD50 and NBS1 [17]. The complex entity supports a nested structure, namely, a complex can be built from sub-complexes.

##### d. Small molecule

This entity enables the modeling scheme to include descriptions of regulations involving small signaling molecules such as GTP, cAMP, Ca^++^, etc. Signaling molecules in SPIKE are identified using their ID in the ChEBI DB [18].

In contrast to genes/proteins and small molecules, no controlled nomenclature is available for designating families and complexes. For these entities we use the most common name in the literature.

#### Relations

The second type of object in our modeling scheme is *relations*. The scheme defines three types of relations:

##### a. Regulation

Every regulation in SPIKE is defined by a triplet of the following simple form: Source, Regulatory effect, and Target. For example, "ATM activates TP53", and "CDKN1A inhibits the CDK2-CCNE complex". A regulatory interaction can be defined between any two biological entities, or between a biological entity and another regulation. A regulation can have one of three regulatory effects: "promote/activate", "inhibit/repress", or "unknown". Each regulation (defined by its source, target and effect) is associated with several attributes: the biochemical mechanism by which it is driven (e.g., phosphorylation, transcriptional regulation, ubiquitination), one or more supporting references, quality level and status flag for quality control (see below), and the submitter/data source.

**Figure 3:**
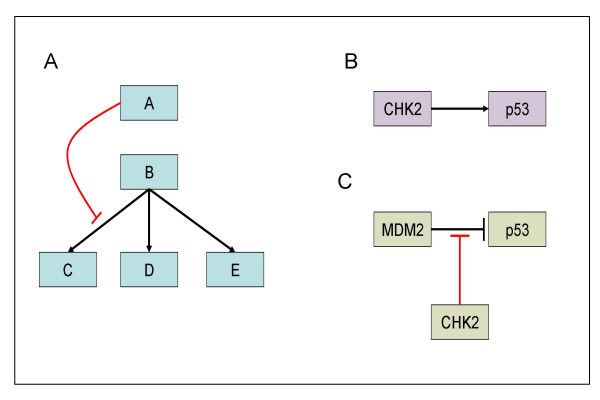
**Regulation as target of another regulation**. (A). The ability to define a regulation as a target of another regulation is helpful in cases where the effect of regulator A on target B is transmitted to some but not all targets of B. In this schematic example, A specifically inhibits the B-mediated activation of C. (B). A schematic representation of p53 activation by CHK2 [31]. (C). A more specific representation: here, the information that the activation of p53 is achieved by CHK2 interfering with the inhibition of p53 by MDM2 is explicitly represented. This interference is achieved by CHK2 phosphorylation of p53 [31], and therefore the '*physical target*' attribute of this regulation is p53.

The fact that our scheme allows one regulation as the target of another enhances its expressivity. First, it enables us to describe regulations that affect only a subset of downstream regulations emanating from an entity, as illustrated in Figure 3A. Second, it improves the specificity of the description, as demonstrated by the comparison between Figure 3B and Figure 3C. For regulations that act on other regulations, an additional attribute is added: the *physical target*, which specifies the physical entity on which the biochemical interaction is exerted (see Figure 3C).

##### b. Interaction

Much data exist on pairs of proteins that are known to physically interact, but whose regulatory effect is not yet understood. *Interaction *relations represent such physical interactions, which, unlike regulations, are symmetrical rather than directional. Each interaction is defined by the two participating proteins, and has the following attributes: the experimental method used to identify it (e.g., Y2H, co-immunoprecipitation), supporting reference(s), quality and status flags, and the submitter/data source.

##### c. Containment

This type of relation is used to define the relationship between families/complexes and the members they contain.

#### Limitations

SPIKE's modeling scheme is intentionally simple, facilitating easy input to the DB by many end-users. Despite its simplicity, the scheme can describe most key aspects of signal transduction pathways. However, at this stage it deliberately does not account for metabolic reactions, splice variants, cell types and cellular compartments, or for any quantitative aspects of regulations. It also deliberately avoids distinction between a gene and its RNA and protein. SPIKE focuses on regulatory effects maintained between components within signaling networks. We found that for our goals it is not essential to explicitly model the layer in which the regulatory effect is exercised (i.e., transcriptional regulation, post-translational modification, etc.). However, information on the specific biochemical mechanism is stored in the DB, being one of the attributes attached to each regulation. While SPIKE's modeling scheme can accommodate data on any signaling pathway from any organism, at present the implemented DB supports data obtained only in human cells (as genes are associated with the human Entrez-Gene ID and are designated using their HGNC official symbol).

Despite these limitations, SPIKE's design is flexible enough to allow its adjustment to support many of the above features in the future, as biological knowledge expands and standard ontologies and nomenclatures become widely accepted.

## Results

### The Database

SPIKE DB contains data from three sources: 1) relations uploaded manually by SPIKE curators and users; 2) relations imported en masse from external signaling pathway DBs (we have already imported data from KEGG [19], Reactome [6] and IntAct [20]). 3) Wide-scale protein-protein interaction datasets that resulted from high-throughput screens in human cells. Currently, the datasets reported by Stelzl et al. [21], Rual et al. [22], Lim et al [23] and Ewing et al. [24] are incorporated. Table 1 summarizes the current status of the DB.

**Table 1:** Current status of SPIKE DB (as of November 2007).

**Quality Level**	**No. of Regulations**	**No. of Interactions**	**No. of Containments**
1	652	7	363
2	17	0	0
3	421	0	521
4	41	21832	906

Total	1,131	21,839	1,790

**Figure 4:**
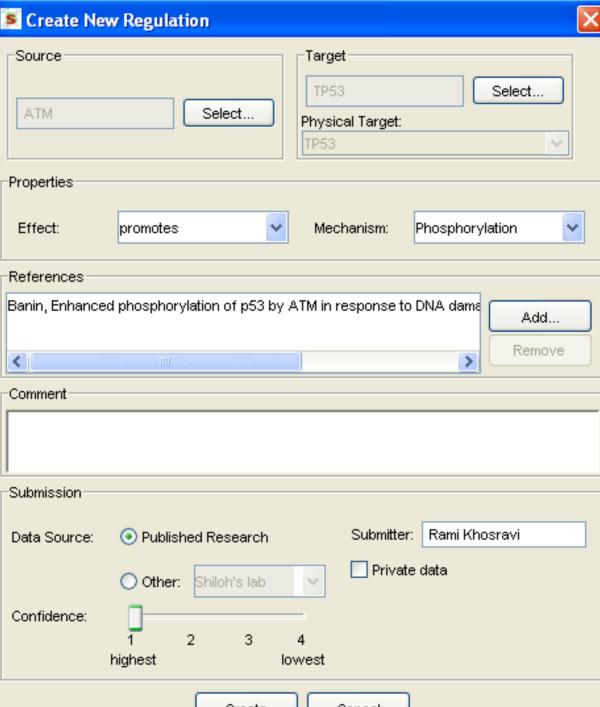
**Adding new information to SPIKE DB**. Submission forms allow privileged users to upload data to the DB. Here, the form for submission of a new regulation is shown. The user specifies the source, target and effect of the regulation. Additional regulation attributes include the biochemical mechanism, one or more supporting references, the submitter, and the quality level ascribed to the data. Submitted data can be flagged as private, in which case the data will be visible only to the user who uploaded it.

SPIKE defines a user hierarchy of four levels: guest, user, curator and admin. Users at the second level and above are privileged to upload new relations to the DB (Figure 4). A privacy mechanism was implemented that allows users to mark as 'private' entries submitted to the DB. Such entries can be accessed only by the laboratory that submitted them. This feature is used for including data in the pre-publication phase. Curated data uploaded manually to date have focused mainly on pathways related to DNA damage responses, cell cycle regulation, apoptosis, innate immunity and stress responses. Data loaded en masse from external KBs and high-throughput datasets cover all aspects of cellular physiology.

The issue of data quality is addressed by several means. First, only privileged users are allowed to upload data to SPIKE DB. Such a privilege is granted only to labs with established expertise in their research field. Second, each submission is assigned a quality level and a status flag. The quality level is set by the user who uploads the regulation and reflects the reliability he/she ascribes to it; in general, relations derived from highly focused biochemical studies are assigned high quality, while those derived from high-throughput experiments are assigned low quality. The status flag is set to 'Draft' at the time of submission. SPIKE designated curators review all new submissions, decide whether to accept, edit or reject them, and set the status flag accordingly.

The current version of SPIKE employs a decentralized DB architecture that supports automatic database synchronization. A local copy of the DB is installed along with the software in each research lab, and these DBs are periodically synchronized with the central DB. A detailed description of SPIKE DB synchronization mechanism is provided in Appendix A. Having local installation of SPIKE DB ensures a genuine and full support for private data. Data are exchanged between the local and central DBs using the SPIKE XML schema that we defined for this purpose (Appendix C). Additionally, the SPIKE XML serves as the primary import/export format in which all the information stored in the SPIKE DB is available. Consequently, this information can be electronically manipulated by any interested party and may be readily incorporated into other external KBs. To facilitate data exchange, we implemented a program that converts SPIKE data to the standard BioPAX format (BioPAX level 2 [25]). The content of SPIKE DB is available in this format from our website. However, due to differences in the representation, BioPaX cannot fully convey all the information stored in SPIKE XML.

### The visualization module

**Figure 5:**
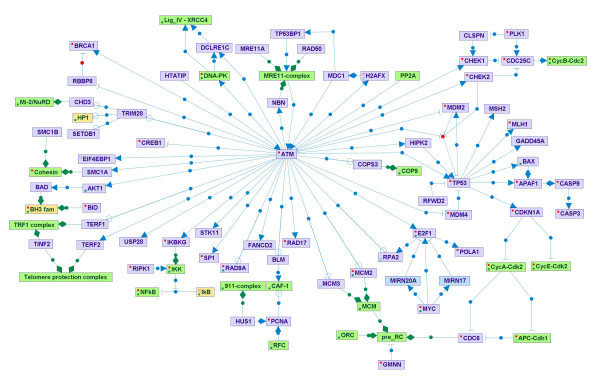
**SPIKE visualization of signaling networks**. The different types of biological entities are represented in SPIKE maps by nodes of different colors: violet nodes correspond to protein-coding genes, turquoise nodes to non protein-coding genes (e.g., micro-RNAs, rRNAs, tRNAs), green nodes to protein complexes, and yellow nodes to protein families. Small molecules are displayed in orange (not included in this map). Blue directed edges represent regulations: arrows correspond to activation, T shape edges to inhibition, and open circles denote regulations whose effect is still not clear (e.g., ATM was reported to phosphorylate MCM2, but the effect of this modification is not known yet). Blue undirected edges represent protein-protein interactions (not included in this map). Green edges represent containment relations between nodes (e.g., a complex and its components). Red and green dots within a node indicate that not all regulation and containment relations stored in the DB for the node are displayed in the map. This map represents the ATM-regulated network which is set off by the cell in response to double-strand breaks in the DNA.

The pathway visualization module allows dynamic network representation of selected biological data stored in the DB and gradual navigation through it. Researchers can manipulate the network map interactively, control its layout, expand or collapse selected segments, and retrieve further information on both biological entities and regulations. SPIKE's four types of biological entities are displayed in the maps using nodes of different colors (Figure 5). Although the modeling scheme does not distinguish between a gene and its RNA and protein products, given the growing data on regulatory roles played by microRNAs in various signaling pathways, protein-coding and non-coding genes are displayed using different colors. The three types of relations (regulations, interactions and containments) are denoted by distinct types of edges (Figure 5).

**Figure 6:**
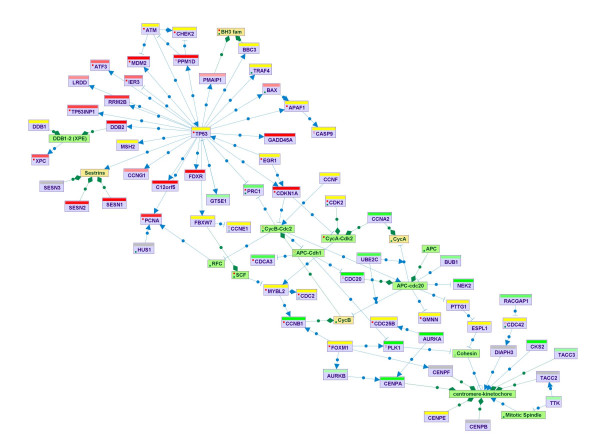
**Superposition of '*omics*' data on maps**. By allowing the user to superimpose functional genomic or proteomic data on pathway maps, SPIKE facilitates improved interpretation of such datasets. The color of the bar above each gene's node indicates its response. Upregulated genes are shades of red: the darker the red the greater the fold of induction. Similarly, downregulated genes are in shades of green. Genes whose expression was not changed are yellow, and genes for which data are not available (e.g., genes not present on the microarray) are grey. In this example, superimposing gene expression data measured in human lymphocyte cells exposed to ionizing radiation on the signaling map shows the activation of the p53 network (top-left part of the network) and the down-regulation of genes that function in the cell cycle G2-M phase transition (bottom-right part of the network). Superimposing the data on this map clearly shows that a large fraction of the p53 network was activated in the analyzed condition. SPIKE also allows superimposition of clustering data: any partition of the genes into groups (e.g., according to GO annotation, cellular compartment, clustering, or user-specific definitions) can be viewed on the pathway map. The color scheme can be adjusted by the user.

Salient features implemented in SPIKE's visualization module include:

• Dynamic navigation and expansion of maps. Double clicking on a node leads to the addition to the map of all its direct neighbors meeting the quality-level threshold set by the user.

• Retrieving further information on genes and regulations. Clicking on a gene's pop-up menu opens its Entrez-Gene web page. Clicking on an edge's pop-up menu allows opening abstracts of the supporting citations from PubMed. In addition, a properties description panel displays more details about each object, in addition to the information supplied in the map itself (e.g., status, quality and submitter data for regulations; full official designation and description for nodes).

• Data filtering. A filtering mechanism allows users to display only data filtered according to different parameters, such as status flag, quality level, data source, and data owner.

• Graph layout. Automatic and manual layout mechanisms can be used to facilitate organization of the maps. Edges can also be moved and manipulated by the user to improve the layout of the map.

• A 'recycling bin' enables users to temporarily hide specific nodes and edges in order to improve clarity of the displayed maps.

• Superposition of *omics *data on the maps. This utility facilitates the biological interpretation of microarray and other high-throughput measurements. For example, coloring maps according to gene expression levels might point to pathways that are active in the analyzed dataset (Figure 6). SPIKE's node coloring mechanism is very flexible and allows the coloring of nodes according to any node partition defined by the user (e.g., GO categories or clustering results).

• Maps built and laid out by the user can be saved and reloaded for further analysis and manipulation. The map files are saved using the SPIKE XML format. This also allows exchange of detailed signaling maps among SPIKE users.

We have built network maps for several key cellular signaling networks, including maps for the p53 and ATM networks, cell cycle, apoptosis, and MAPK and TLR signaling. The maps are posted on the SPIKE website. Importantly, these are not static maps: they are linked to the SPIKE DB, so when loaded by the user they can be further expanded (by double-clicking on selected nodes). In addition, updates to the DB are automatically reflected in the map (the existence of new regulations that involve any node in the map and that are not displayed in it will be indicated by a red dot within the node). This ensures that the maps posted on the site are continuously synchronized with the DB. SPIKE users can join the collaborative effort, building signaling maps for pathways that are in the focus of their research, and contributing these maps to the community by posting them on the SPIKE web site.

### The algorithmic engine

SPIKE's algorithmic engine is designed to include various graph-theory algorithms that analyze the network, characterize its topological properties, integrate *omics *data to identify functional modules in the network, and suggest novel regulatory interplays between components. At present, we have implemented three basic utilities:

#### • Path finding

**Figure 7:**
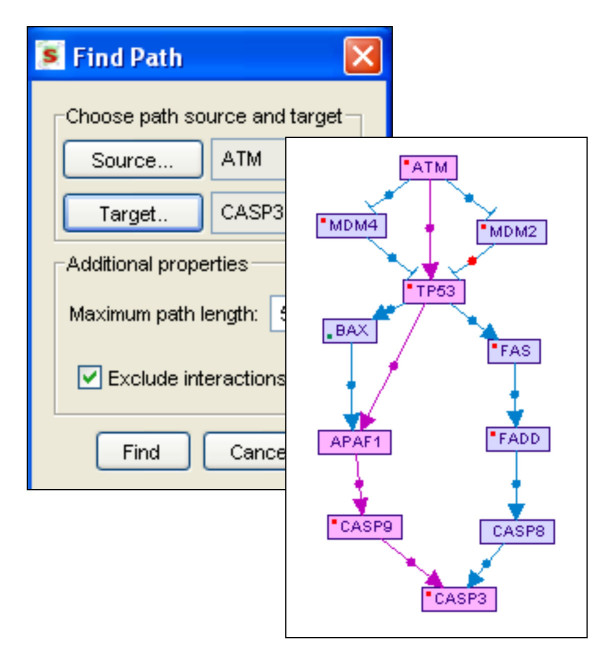
**Path finding**. The user specifies the source, the target and the maximal number of edges in a path connecting them. By default, un-directed edges (that is, protein-protein interactions) are excluded from the analysis. In this example, pathways linking ATM to CASP3, a major effector of apoptosis, are sought. All paths that meet the length constraint are displayed with the shortest paths highlighted.

This utility finds and displays all direct paths, up to a pre-defined maximal length, that connect source and target nodes specified by the user (Figure 7). This utility supports also undirected edges (interactions). However, by default, undirected edges are excluded from this analysis as such links are much less informative than directed ones. The user can choose whether to include or exclude interactions (which are usually of lower quality level than the regulations in SPIKE DB).

#### • Interconnections within a set of genes/proteins

**Figure 8:**
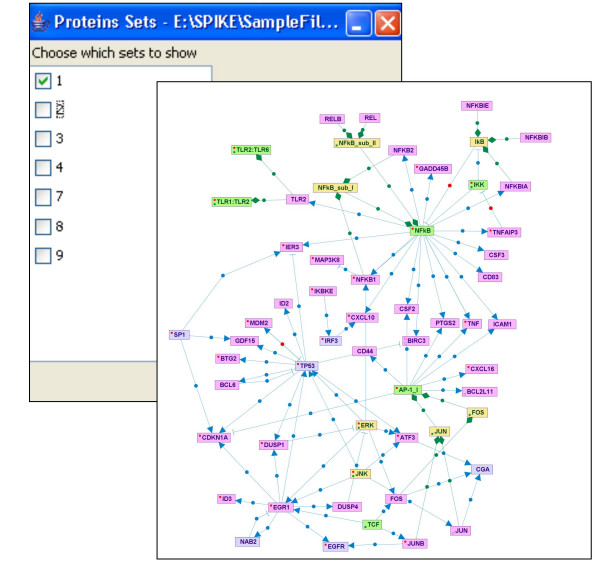
**Interconnections within a set of genes/proteins**. Interconnections among nodes within a specified target set of genes or proteins (e.g., sets obtained by cluster analysis applied to gene expression microarray data) are displayed. This figure shows the interconnections within a cluster of genes that were induced by LPS treatment, including single intermediate nodes not contained in the original cluster. Nodes corresponding to genes contained in the input cluster are highlighted in pink.

Given target sets of genes/proteins provided by the user (e.g., gene clusters identified by analysis of gene expression data), this utility displays all the direct connections among nodes within each set (with an option to allow connection via a single intermediate node not included in the input set) (Figure 8).

#### • Enriched maps

**Figure 9:**
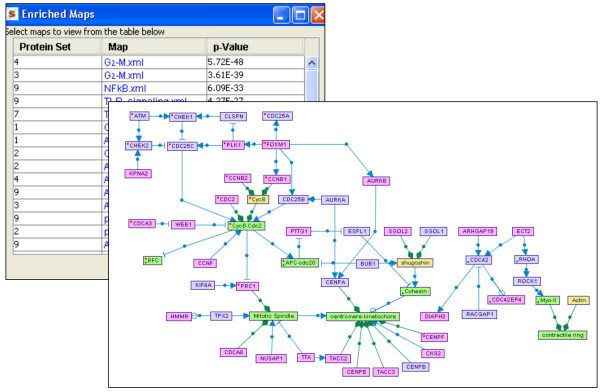
**Enriched maps**. Signaling maps enriched for nodes included in target sets of genes/proteins are sought. Maps that are significantly enriched (beyond a specified p-value threshold) are listed on the results table. Clicking on a map's link in this table opens the enriched map, in which nodes contained in the original input set are highlighted.

Given target sets of genes/proteins uploaded by the user, the utility searches for signaling maps enriched for nodes included in one of the target sets. Enrichment scores are calculated using the hypergeometric distribution and are computed with respect to a given background set (e.g., all the genes present on a microarray platform). Maps that are significantly enriched (beyond a specified p-value threshold) are listed in the results table. Clicking on a map's link in this table opens the enriched map, in which target set nodes are highlighted (Figure 9).

### Installation

SPIKE can be downloaded from [26]. It has two modes of installation: a stand-alone tool (evaluation mode) and a community tool (standard mode). Installing the evaluation mode (which is based on the Java Web-Start technology) is straightforward: It requires the execution of a single file, and installation is completed by a few clicks. It also provides a mechanism for automatic software upgrades and maintenance. This installation is currently limited to Microsoft Windows 2000/XP OS and requires ad-hoc procedures for synchronization of data with the central SPIKE DB.

A research group that wishes to join the SPIKE community should fill-in an online form available at SPIKE website and have the tool locally installed in its "standard installation" mode. The standard mode of installation grants the user full functionality of the tool. Mainly, it supports synchronization between the site and the central DB and can serve several users on different machines within one internal network site (typically, several users within a research lab and several research labs in the same institute). It requires installation of the MySQL DB server on one machine on the site. Detailed installation instructions are provided on the website. This installation mode allows data submitted at the site to be shared with the entire SPIKE community (while retaining the option to hide some private data), and enables that site to benefit from the data submitted by other sites. The standard installation supports Windows, UNIX and Mac OS.

## Conclusion

SPIKE provides the research community with a powerful integrated KB of signaling pathways in human cells. The tool helps researchers integrate, visualize and interpret existing and novel information on cellular networks. It can be of great utility both for labs whose research is focused on specific processes and networks (e.g., p53 network), and for '*omics*' labs that strive for comprehensive, systems-level delineation of cell functioning.

Gene/protein-centric labs can utilize SPIKE as a central repository for regulations and interactions involving genes, proteins and processes that are in the focus of their research, and greatly benefit from data uploaded by other labs in the same research field. As a pilot case, at present we are focusing our curation effort on signaling pathways that are induced by DNA damage (including DNA repair, cell cycle checkpoints, cell death and general stress responses), and are building a community of SPIKE users in the DNA damage research field. Any biologist in this community can find SPIKE very useful in addressing simple queries such as 'which proteins are directly regulated by p53?', 'is there a pathway that connects two genes whose knockdown results in a similar hyper-sensitivity to DNA damage?'. In parallel, we encourage and support the establishment of additional sub-communities that cover other aspects of cellular physiology.

Labs that carry out wide-scale 'omics' experiments can utilize SPIKE's analytic and visualization features to mine meaningful biological hypotheses out of the data (e.g., by using map enrichment tests or the interconnection utility), and present them in lucid, aesthetic network graphs. The flexible coloring schemes implemented in SPIKE make it very powerful for this task. For example, using SPIKE we built network maps in which gene nodes were colored according to the kinetics in which they were induced in the dataset that we analyzed and according to regulatory-elements that we identified in their promoter regions [27,28].

SPIKE is under continuous development and we plan to enhance several functionalities in future versions. It will be upgraded to a web-based system that will enable its use over the web without local installation. It will be developed into a multiple-species KB. Visualization capabilities will be enhanced by adding hierarchical and other graph layout mechanisms. We are currently implementing an interface between SPIKE and our EXPANDER package for analysis of microarray data [29] aimed at creating one streamlined process for handling such data, beginning in the very early preprocessing stage of data-normalization and ending with integrating the results with knowledge on signaling networks. We are also using high-throughput results to develop algorithms for the identification of 'connected modules' and 'hot-spots' in the network (for example, identification of sub-regions that are significantly dense in genes/proteins that show some property of interest, see for example our recent work [30]). In parallel, we will continue to populate the SPIKE DB, supervise its quality, and build additional maps for pivotal signaling pathways.

## Availability and requirements

**Project name: **SPIKE

• **Project home page: **

• **Operating system(s): **Windows, UNIX or Mac.

• **Programming language: **Java

• **Other requirements: **Java 1.4 or higher, MySQL DB server

• **License: **free for non-commercial users

• **Any restrictions to use by non-academics: **license needed

## Authors' contributions

RE, YS and RS designed the functionality of tool and coordinated its development. RV, NA, IU, IZ and JA developed the system architecture, wrote the code and tested it. NO, GS and RB implemented an earlier version of the system. RE also populated the database and performed beta testing. GM and MW are the principal curators of SPIKE. All the authors have read and approved the final version of the manuscript.

## Appendix A – DB synchronization

SPIKE's DB synchronization module makes it possible for remote sites to receive and send updates from/to the central DB. Below we describe the logic of DB synchronization as implemented for remote sites and for the central site.

A snapshot of the central DB saved in XML format on the SPIKE web site, and is updated periodically. Upon initiation of the synchronization process at the remote site, SPIKE downloads the latest version of the central DB from the web site and compares it with the local DB. All entities in the SPIKE DB are tagged with an internal accession ID. This ID is unique, yet it is possible that the same object will temporarily have different accession IDs in different DBs (e.g., the same regulation was defined by several users at different sites). Therefore, objects in the central snapshot that match those present in the remote DB are first sought using SPIKE accession ID, and then by searching for objects with identical defining components. For example: families and complexes are defined by the set of their *genes/proteins *members; a regulation is defined by its *source*, *target *and *effect*.

The synchronization process first handles entities and relations that are present in both the local and central DBs, starting with matching *biological entities *and proceeding to matching *relations*. The tool identifies differences between matching objects (such as: the quality level of a regulation was changed at the central site; a supporting reference for a regulation was added at the remote site; a member of a family was deleted at the central site) and handles these differences by the following logic: Each DB change that is applied by a remote site is attached with a *change-id*, and for each remote site there is a record (*last-review-change-id*) for the last change that was already reviewed by a curator at the central site. Then, if an object that is present in both DBs is attached with a *change-id *that is smaller than the *last-review-change-id *(meaning that either the object was changed by the central or other remote sites, or that a change done by the remote site was rejected by the central site), then the object's version of the central DB prevails and overwrites the local version. Otherwise (*change-id *is higher than the *last-review-change-id*), the local version of the object prevails and remains as is.

Following the handling of objects that are present in both DBs, the procedure handles objects that exist in only one of them: objects that exist only in the local DB remain as they are and objects that exist only in the central snapshot are added to the local DB. At the end of the synchronization process, the application provides the user with a summery of the updates made to the local database and creates an XML snapshot of it, which is sent to SPIKE central site. Importantly, data uploaded at the remote site and flagged as private are excluded from the snapshot, ensuring the confidentiality of such data. A curator at the central site will review the snapshot sent by a remote site and integrate it into the central DB.

The logic of DB synchronization carried out at the central site is similar to the one of the remote sites with the major difference that while at remote sites local DB changes due to synchronization are exerted automatically, at the central site all DB changes are presented to and manually reviewed by a curator (Additional file 1). These changes include differences between objects that are present in both DBs and whose remote *change-id *is higher than the *last-review-change-id *of that remote site, or objects found only in the remote DB. The curator examines these differences and decides which to approve (possibly after some editing) and which to reject. A decision to reject may involve a discussion with the author of the change in order to understand the cause of disagreement. The *status flag *of changes approved by the curator is set from *draft *to *approved*. The central database is updated by the application according to the curator's decisions. When the update is completed, the *last-review-change-id *parameter of the corresponding remote site is updated, and a new snapshot of the central database is posted on the web site in XML format.

We have thoroughly tested the synchronization module and implemented an automatic benchmarking suit that examines proper functionality of the module both at remote and the central sites under different DB change scenarios (See Appendix B). A typical DB synchronization takes approximately 5 minutes on a PC with at least 0.5 GB RAM.

## Appendix B – Benchmarking of DB synchronization

We have implemented an automatic benchmarking suit that examines proper functionality of the DB synchronization module both at remote and the central sites under different DB change scenarios as listed below.

### Remote side

The tested scenarios for the remote site are:

1. Addition of a group by the remote-site – should not be affected by synchronization

2. Deletion of a group by Central – should be removed from the remote site after synchronization

3. Addition of a group by central – should be added to the remote site after synchronization

4. Editing of an existing group locally – should not be affected by synchronization

5. Editing of an existing group by Central – should be updated at the remote site after synchronization

6. Editing of an existing group both locally and by Central – should not be affected by synchronization

7. Same tests for regulations and interactions.

### Central side

The tested scenarios for the central site are (the tests assume that the curator approved the change exerted by the remote site):

1. A group deleted at the central site still exists in the remote site – the group should not be restored by synchronization

2. Addition of a group by Central – should not be affected by synchronization

3. Addition of a group by the remote site – should be added to the central site after synchronization

4. Editing of an existing group by the remote site – should be updated at the central site after synchronization

5. Editing of an existing group by Central – should not be affected by synchronization

6. Editing of an existing group both by a remote site and by Central – should accept remote changes

7. same tests for regulations and interactions

## Appendix C – SPIKE XML

As the information stored in the SPIKE database is fundamentally different from that stored in reaction-based database, the widely accepted BioPax and SBML formats were not found to be suitable for conveying SPIKE pathways. We thus developed a specialized XML schema for describing a SPIKE pathway. Importantly, the same XML format is used for all communications between SPIKE sites and for storing pathway maps. Also, the format is extremely simple and can be easily used for converting information to and from other formats. Examples of XML files can be found on the SPIKE website (see Availability and requirements section for url, follow the Maps link therein).

On the top level of the XML, <**SpikeDatabase**> conveys general information on the version of the database from which the pathways were extracted. On the next level the following information is encapsulated into three blocks:

• <**SiteBlock**> contains information about SPIKE sites, intended for internal use of SPIKE. Each site is represented by a single <**Site**> tag

• <**BuildingBlock**> contains information about the basic entities in SPIKE.

• <**RegulationBlock**> contains information about the regulations contained in the pathway.

• <**InteractionBlock**> contains information about the interactions contained in the pathway.

The <**BuildingBlock**> contains entities referring to single genes/proteins or gene/protein groups:

• A <**Gene**> entity refers to a gene/protein and is linked to an EntrezGene identifier through an <**XRef**> tag. Information about the graphical properties of an entity, its description and aliases are given in the <**Display**>, <**Description**> and <**Alias**> tags, respectively.

• A <**Group**> entity contains information about the two groupings supported by SPIKE: complexes and families. These two groupings are distinguished by the **type **attribute of the entity. The members of the group are described in <**Member**> entities, each referring to an identifier of another entity from the <**BuildingBlock**>.

The <**RegulationBlock**> contains <**Regulation**> entities describing regulations within the pathway. Recall that regulations in SPIKE are directed events (e.g., A activates/inhibits B) as opposed to mere interactions (which are found in the <**InteractionBlock**>). The constituents of the regulation are referred to by the <**Source**>, <**Target**> and <**PhysicalTarget**> entities. Note that while the target of a regulation can be any entity from the <**BuildingBlock**> or from the <**RegulationBlock**>, the physical target and the source are always entities from the <**BuildingBlock**>. Literature support for each regulation entity is given in a <**Reference**> entity.

The <**InteractionBlock**> is generally similar to the <**RegulationBlock**>. It consists of <**Interaction**> entities, describing an undirected connection between a pair of entities from the <**BuildingBlock**>, which are referred to by the <**ProteinA**> and <**ProteinB**> entities.

As an example, below we give SPIKE XML statements for the three cartoon cases shown in Figures 3A–3C. For clarity, we include only the core parts of SPIKE XML format and leave out additional attributes, such as links to other databases, visual settings and information on the source and quality level of the regulations.

Figure 3A:

   <Regulation id="B→C">

<Source ref="B"/>

<Target ref=" C"/>

<PhysicalTarget ref="C"/>

   </Regulation>

   <Regulation id="B→D">

<Source ref="B"/>

<Target ref=" D"/>

<PhysicalTarget ref="D"/>

   </Regulation>

   <Regulation id="B→E">

<Source ref="B"/>

<Target ref="E"/>

<PhysicalTarget ref="E"/>

   </Regulation>

   <Regulation id="A--| (B→C)">

<Source ref="A"/>

<Target ref="B→C"/>

<PhysicalTarget ref="B"/>

   </Regulation>

Figure 3B:

   <Regulation id="CHK2--| p53">

<Source ref="CHK2"/>

      <Target ref=" p53"/>

      <PhysicalTarget ref=" p53"/>

   </Regulation>

Figure 3C:

   <Regulation id="MDM2--| p53">

<Source ref=" MDM2"/>

      <Target ref=" p53"/>

      <PhysicalTarget ref=" p53"/>

   </Regulation>

   <Regulation id="CHK2--| (MDM2--| p53)">

<Source ref="CHK2"/>

      <Target ref="MDM2--| p53"/>

      <PhysicalTarget ref="p53"/>

   </Regulation>

## Supplementary Material

Additional file 1 **Curator's report for DB synchronization**. When synchronizing the central DB with a snapshot of a remote site DB, all DB differences are reported to the curator at the central site in such a report table. The curator manually reviews it and decides which changes to accept and which to reject. Click here for file
