# Supplementary figures and images for: SPIKE – a database, visualization and analysis tool of cellular signaling pathways
Source: BMC Bioinformatics. 2008 Feb 20;9:110. doi: 10.1186/1471-2105-9-110 (PMC2263022; doi:10.1186/1471-2105-9-110)

## Slide 1
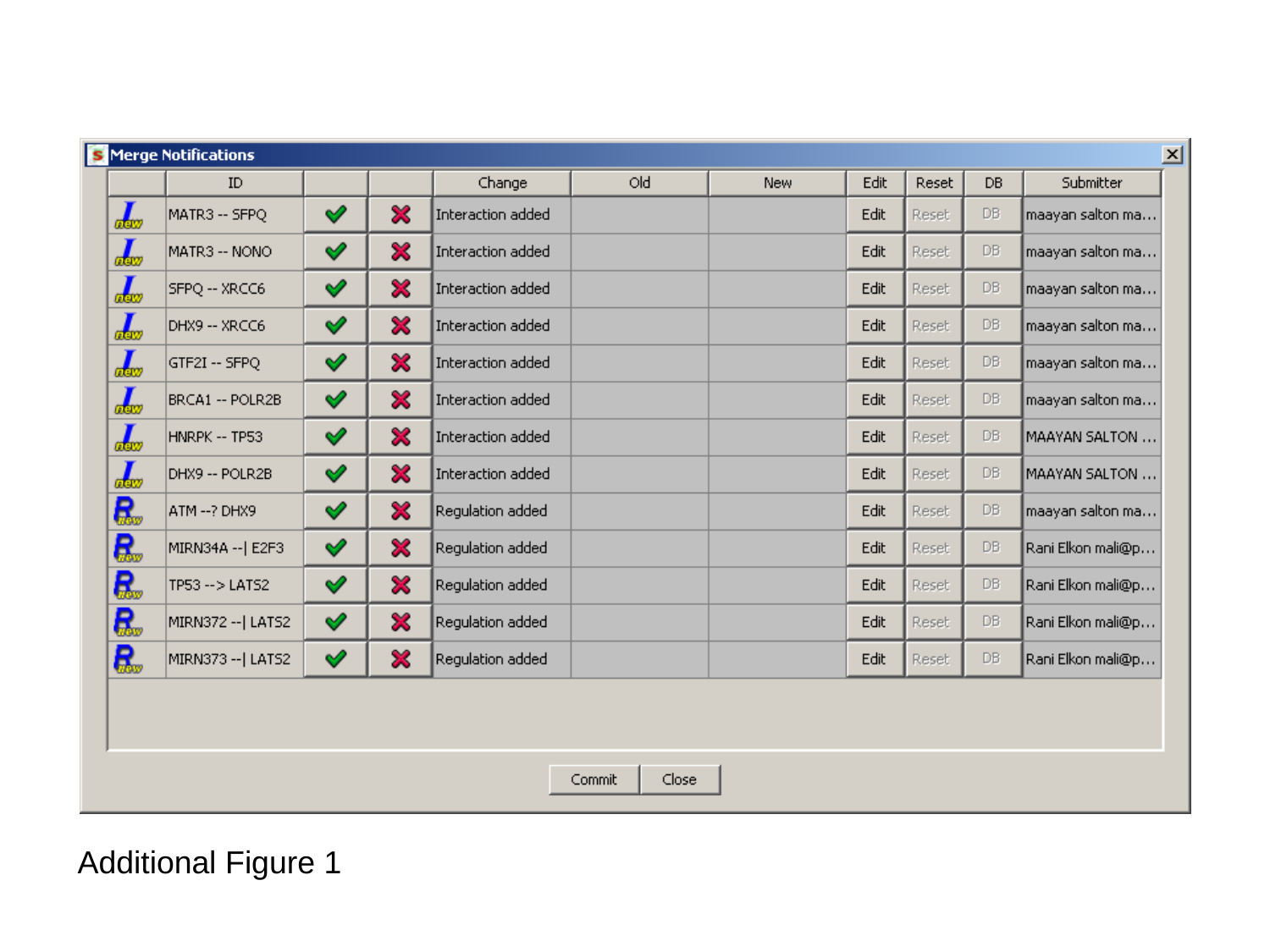

Additional Figure 1

Supplement: Additional file 1 — Curator's report for DB synchronization. When synchronizing the central DB with a snapshot of a remote site DB, all DB differences are reported to the curator at the central site in such a report table. The curator manually reviews it and decides which changes to accept and which to reject. [file 1471-2105-9-110-S1.ppt]
